# Mendelian randomization analyses reveal causal relationships between the human microbiome and longevity

**DOI:** 10.1038/s41598-023-31115-8

**Published:** 2023-03-29

**Authors:** Xiaomin Liu, Leying Zou, Chao Nie, Youwen Qin, Xin Tong, Jian Wang, Huanming Yang, Xun Xu, Xin Jin, Liang Xiao, Tao Zhang, Junxia Min, Yi Zeng, Huijue Jia, Yong Hou

**Affiliations:** 1 College of Life Sciences, University of Chinese Academy of Sciences, Beijing, 100049 China; 2 BGI-Shenzhen, Shenzhen, 518083 China; 3 James D. Watson Institute of Genome Sciences, Hangzhou, 310058 China; 4 Department of Biology, University of Copenhagen, Universitetsparken 13, 2100 Copenhagen, Denmark; 5 School of Medicine, The First Affiliated Hospital, Institute of Translational Medicine, Zhejiang University, Hangzhou, China; 6 Center for Healthy Aging and Development Studies, National School of Development, Raissun Institute for Advanced Studies, Peking University, Beijing, China; 7 Greater Bay Area Institute of Precision Medicine (Guangzhou), Fudan University, Shanghai, China

**Keywords:** Genetic association study, Genomics

## Abstract

Although recent studies have revealed the association between the human microbiome especially gut microbiota and longevity, their causality remains unclear. Here, we assess the causal relationships between the human microbiome (gut and oral microbiota) and longevity, by leveraging bidirectional two-sample Mendelian randomization (MR) analyses based on genome-wide association studies (GWAS) summary statistics of the gut and oral microbiome from the 4D-SZ cohort and longevity from the CLHLS cohort. We found that some disease-protected gut microbiota such as Coriobacteriaceae and *Oxalobacter* as well as the probiotic *Lactobacillus amylovorus* were related to increased odds of longevity, whereas the other gut microbiota such as colorectal cancer pathogen *Fusobacterium nucleatum, Coprococcus*, *Streptococcus*, *Lactobacillus*, and *Neisseria* were negatively associated with longevity. The reverse MR analysis further revealed genetically longevous individuals tended to have higher abundances of *Prevotella* and *Paraprevotella* but lower abundances of *Bacteroides* and *Fusobacterium* species. Few overlaps of gut microbiota-longevity interactions were identified across different populations. We also identified abundant links between the oral microbiome and longevity. The additional analysis suggested that centenarians genetically had a lower gut microbial diversity, but no difference in oral microbiota. Our findings strongly implicate these bacteria to play a role in human longevity and underscore the relocation of commensal microbes among different body sites that would need to be monitored for long and healthy life.

## Introduction

Population aging is accelerating, and it is of great significance to identify the biological factors that affect aging and how to extend life to achieve a healthy and long life. The heritability of a human’s lifespan is reported to be only 25%^1,2^, which means the human lifespan is not only determined by the genome but also affected by both the human external environment and internal environment (microbiome) as well as their interactions^3,4^. Increasing evidence indicates that there is a link between gut microbes and longevity^5–9^. A study of Italian centenarians (99–104 years old) and ultracentenarians (105–109 years old) showed that there is a different core flora that decreases with aging dominated by Ruminococcaceae, Lachnospiraceae, and *Bacteroides*^6^. Another independent study of Chinese centenarians showed that aging is negatively related to abundances of *Bacteroides vulgatus, Ruminococcus sp.5139BFAA,* and *Clostridium* sp. *AT5* in the gut^7^. These studies have reported that some specific intestinal microbes are significantly associated with longevity, however, the causal relationships between them remain unexplored. In addition, the oral microbiome influenced not only oral but also systemic health and disease^10–12^. Few studies have focused on the associations between the oral microbiome and longevity. Therefore, it is particularly important to explore in detail which specific intestinal or oral microbes link to longevity, and to explore the functions of these intestinal microbes, to achieve aging intervention and prolong lifespan.

Mendelian randomization (MR)^13^ has been developed to investigate the potential causal relationship between exposure and outcome by using genetic variants as instrumental variables (IVs) in genetic epidemiology research. Several MR analyses have investigated the biomarkers (genes/proteins/metabolites/diseases) contributing to longevity or lifespan^14–18^. The microbiota influencing longevity remains unknown. Twin-based heritability estimation^19,20^ and increasing microbiome GWAS analyses^21–25^ consistently indicated the genetic influence on gut microbiota. Our recent microbial GWAS analyses have identified genetic determinants for both gut^21^ and oral^26^ microbial features, which enable us to investigate potential causations between the human microbiome and longevity.

Here, we performed bidirectional two-sample MR analyses to dissect the potential causal links between the human microbiome and longevity. Specifically, the forward MR was performed using gut-, salivary-, tongue dorsum- microbiome, and microbial α-diversity from the 4D-SZ cohort (N = 2984) as exposures and longevity from the CLHLS phase I dataset (N = 4477) as the outcome, to identify the microbial features that may affect longevity. The reverse MR was performed using the longevity phase I dataset (N = 4477) as discovery sample exposure and the longevity phase II dataset (N = 6548) as replication sample exposure, together with gut-, salivary-, tongue dorsum- microbiome, and microbial α-diversity as outcomes, to identify the causal effects of genetically predicted longevity phenotype on the microbiota. An overview of the study design and results was presented in Supplementary Fig. 1. Additionally, we performed the main MR analyses in Chinese populations and replicated the MR results with that of in European populations. In this large-scale MR study, we discovered a large number of potential causal correlations between the gut, oral microbiota and longevity, suggesting the human microbiome is a promising alternative for microbiome-based management of human healthy aging.

## Results

### Causal relationships between the gut microbiome and longevity

**Figure 1:**
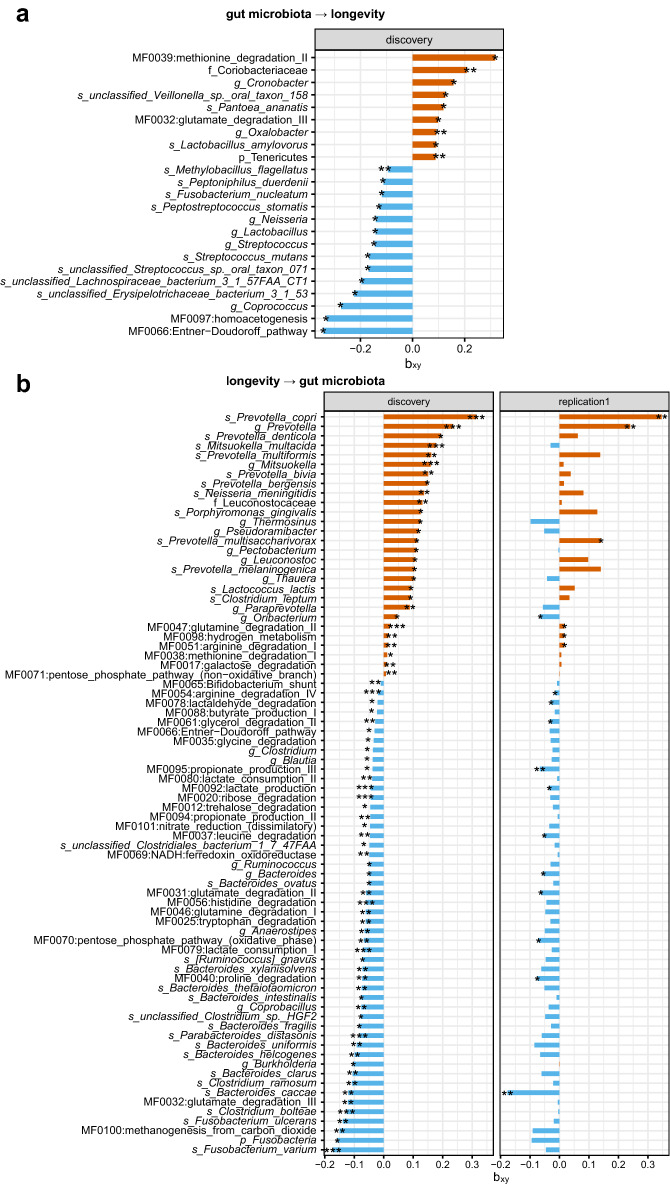
Bidirectional two-sample MR analyses identified causal relationships between the gut microbiome and longevity. (**a**) The barplot represented the causal effects of 23 specific gut microbial features on longevity (*p* < 0.05), as estimated using the GCTA-GSMR approach in the forward MR analysis. The MR effect size estimate (bxy) was plotted. (**b**) The barplot represented the causal effects of longevity on 78 gut microbial features in the reverse MR analysis (*p* < 0.05). Discovery used the longevity phase I dataset (N = 4477) as exposure. Replication used the longevity phase II dataset (N = 6548) as exposure. Both used the gut microbiome dataset (N = 1539) as the outcome. *p < 0.05, **p < 0.01, and ***p < 0.001. s_ denotes species, g_ denotes genus, f_ denotes family, p_ denotes phylum and MF denotes microbial function/module.

We first investigate causal relationships between the gut microbiome and longevity by performing a bidirectional two-sample MR analysis using GWAS summary data of the gut microbiome (500 independent microbial features; N = 1539) obtained from the 4D-SZ cohort^21^, together with longevity GWAS from the CLHLS cohort^27^ (N = 4477) (Supplementary Fig. 1, “Methods” section). Descriptions of samples and cohorts were presented in Supplementary Table 1. In the forward MR, 23 gut microbial features were found to influence longevity (*p* < 0.05; Fig. 1; Supplementary Table 2). Family Coriobacteriaceae (β = 0.21, *p* = 0.008), phylum Tenericutes (β = 0.089, *p* = 0.008), genus *Oxalobacter* (β = 0.09, *p* = 0.0096), microbial function/module MF0039:methionine degradation II (β = 0.32, *p* = 0.014) and species *Lactobacillus amylovorus* (a probiotic^28^ altering body adiposity; β = 0.09, *p* = 0.014) were the top five strongest positively associated with longevity. However, some features like *Methylobacillus flagellates* (β =  − 0.094, *p* = 0.003), Neisseria genus, *Streptococcus* genus and its species (the dental caries pathogen *S. mutans*^29^ and unclassified *Streptococcus* sp. *Oral taxon 071*), and *Fusobacterium nucleatum* (colorectal cancer (CRC) pathogen^30^; β =  − 0.118, *p* = 0.036) were negatively associated with longevity. These causal relationships were robust with *p* < 0.05 in at least one method when the other five two-sample MR tests were performed (“Methods” section), and there was no evidence of horizontal pleiotropy (both *P*_MR-PRESSO.Global.test_ > 0.05 and *P*_MR_egger_intercept_ > 0.05; Supplementary Table 2).

In the reverse MR analysis to use longevity as exposure (N = 4477 with 45 independent IVs; Supplementary Fig. 1), we found longevity was associated with relative abundances of 78 microbial features (*p* < 0.05). Notably, genetically predicted longevous individuals tended to have increased abundances of some features like the genus *Prevotella* and its seven species such as *P. copri* and *P. multiformis*, *Paraprevotella Mitsuokella, and* MF0047:glutamine degradation II, but decreased abundances of other features such as *Bacteroides* and its nine species such as *B. thetaiotaomicron* and *B. uniformis*, phylum Fusobacteria and its species (*F. varium* and *F. ulcerans*), genus *Clostridium* and its species (*C. bolteae*, *C. ramosum*, unclassified *Clostridium* sp. HGF2, and *C. leptum*). In general, *Bacteroides* and *Escherichia* were more prevalent than *Prevotella* after 50 years old^20,31^. *Bacteroides* and *Fusobacteria* were also major taxa that metabolize pectin, implicated in body fat and hormone levels^32^.

**Figure 2:**
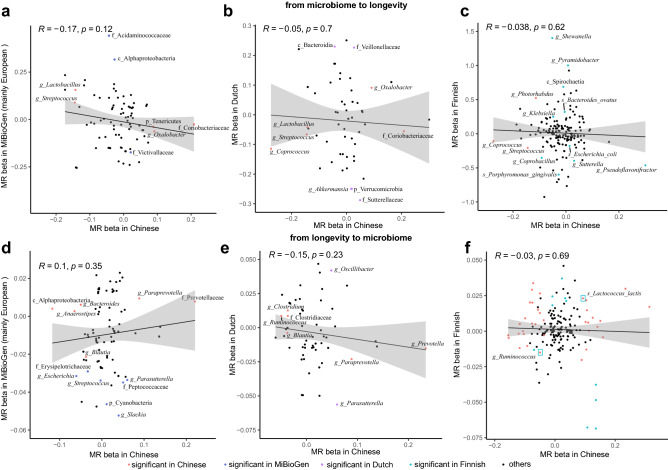
Comparisons of causalities between Chinese and European populations. (**a–c**) represented the causalities from gut microbiome to longevity by comparing the effect sizes of all available causalities between Chinese and MiBioGen (mainly European, (**a**)), Finnish (**b**), and Dutch (**c**), respectively. (**d–f**) represented the causalities from longevity to gut microbiome by comparing the effect sizes of all available causalities between Chinese and MiBioGen (mainly European, (**d**)), Finnish (**e**), and Dutch (**f**), respectively. Spearman test was used with correlation coefficients and *p*-value marked. The red, blue, purple, and green dots indicated the causalities were significant in Chinese, MiBioGen, Dutch, and Finnish, respectively (*p* < 0.05). The black dots indicated the causalities were not significant in either of the two compared populations. The red dots with a green rectangular box in the Dutch panel represented the shared MR by the Chinese and Dutch.

70 of the 78 causal relationships were replicated in the same direction of effect, of which 17 were well validated with p < 0.05 while applying the reverse MR analyses to another independent longevity dataset^33^ as exposure (N = 6548 with 30 independent IVs; Supplementary Table 3), suggesting these causalities were robust. We confirmed the correlation of longevity with increased abundances of *Prevotella* and its species (Fig. 2) but decreased abundances of *Bacteroides* and its species (Supplementary Fig. 3), in line with previous observational studies^34^. Additionally, longevity was related to increased abundances of microbial pathways such as MF0047: glutamine degradation II, MF0098: hydrogen metabolism, and MF0051: arginine degradation I, but the decreased abundances of pathways like MF0092: lactate production, MF0040: proline degradation, F0070: pentose phosphate pathway (oxidative_phase) and MF0095: propionate production III, which were all replicated in the MR analyses when using two independent longevity cohorts as exposure datasets.

### Few overlaps of causalities between the Chinese and European populations

To confirm if the causalities identified in the Chinese were replicated in other populations, we systematically compared the correlations of the gut microbiome and longevity with those of the European cohorts. We performed bidirectional two-sample MR analyses by leveraging GWAS summary statistics of the gut microbiome from MiBioGen (mainly European populations)^22^, Dutch^35^, and Finnish^36^ cohorts, together with the largest meta-analysis of longevity GWAS of European ancestry^37^. Overall, MR results from the MiBioGen, Dutch, and Finnish cohorts (Supplementary Tables 4–6) showed clear differences from those in the Chinese cohort. Beta effects of the causalities available between Chinese and the three European cohorts consistently showed negative correlations, except the only one positive correlation of causality from longevity to microbiome between Chinese and the MiBioGen cohort (Fig. 2; Supplementary Figs. 4, 5).

Despite the overall negative correlations of the causalities between the Chinese and European populations (Fig. 2; Supplementary Tables 4–6), we found two shared causalities: longevity was causally linked to an increased abundance of *Lactococcus lactis,* but the decreased abundance of *Ruminococcus* in both Chinese and Finnish (Fig. 2f). Oral *L. lactis* enriched in healthy people in comparison with RA^10^, could inhibit bacteria such as *Streptococcus*^38^, and could produce GABA^39^. We also observed several overlapped causalities among the three European cohorts. Bacteroidia was positively associated with longevity in the Dutch cohort and so with two *Bacteroides* species (*B. sp003545565* and *B. ovatus*) in the Finnish cohort. Sutterellaceae in the Dutch cohort and *Sutterella* in the Finnish cohort were negatively linked to longevity. We called for further validation of these shared microbiome-longevity interactions and the discovery of more overlapped causalities across populations in the future, given mainly 16s rRNA sequencing classifying into only microbial genera but not species was performed in the European populations.

### Causalities between the salivary microbiome and longevity

**Figure 3:**
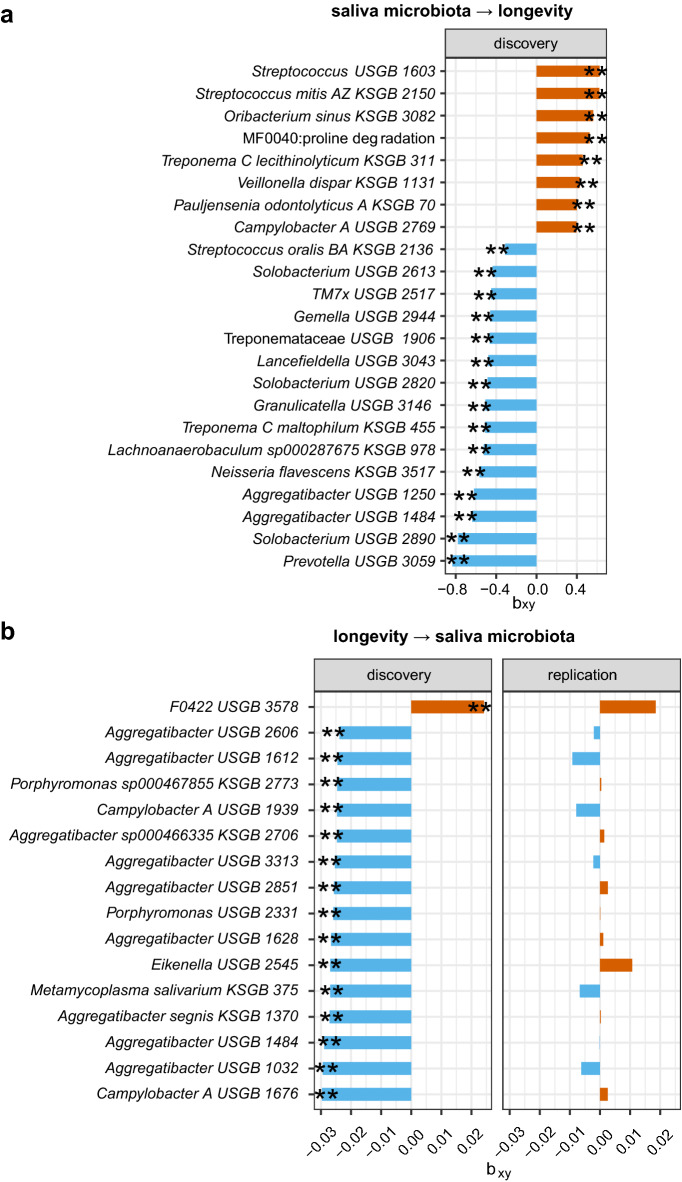
Bidirectional two-sample MR analyses identified causal relationships between the salivary microbiome and longevity. (**a**) The barplot represented the causal effects of 23 specific salivary microbial features on longevity (*p* < 0.01), as estimated using the GCTA-GSMR approach in the forward MR analysis. The MR effect size estimate (bxy) was plotted. (**b**) The barplot represented the causal effects of longevity on 16 salivary microbial features in the reverse MR analysis (*p* < 0.01). Discovery used the longevity phase I dataset (N = 4477) as exposure. Replication used the longevity phase II dataset (N = 6548) as exposure. Both used the gut microbiome dataset (N = 1539) as the outcome. **p < 0.01. Unclassified species-level cluster denotes uSGB, known species-level clusters denote kSGB and MF denotes microbial function/module.

We observed 88 significant associations of the salivary microbiome with longevity (*p* < 0.05; Supplementary Table 7), among which 23 correlations with *p* < 0.01 were presented in Fig. 3a. Several microbes such as *Streptococcus mitis AZ* (β = 0.623, *p* = 0.0012), *Pauljensenia odontolyticus A* (β = 0.399, *p* = 0.0013), *Treponema C lecithinolyticum* (β = 0.458, *p* = 0.0035), and *Streptococcus uSGB 1603* (β = 0.625, *p* = 0.0045) linked to increased longevity odds. *Streptococcus uSGB 1603* reduced risks of uterine fibroids and gastric cancer in the diseases’ MR analysis by integrating with biobank Japan (BBJ) diseases data (Supplementary Table 8). *Oribacterium sinus* which has been reported depleted in the COVID-19 patients^40^ and potentially negatively correlated with congestive heart failure (Supplementary Table 8), exhibited a positive association with longevity. Reversely, some oral microbiota such as *Neisseria flavescens* (β =  − 0.565, *p* = 0.0015), *Lachnoanaerobaculum sp000287675* (β =  − 0.523, *p* = 0.0017), *Solobacterium uSGB 2890* (β =  − 0.780, *p* = 0.0017), *Lancefieldella uSGB 3043* (β =  − 0.477, *p* = 0.0032) decreased longevity odds. *Lachnoanaerobaculum sp000287675* increased the risk of chronic obstructive pulmonary disease, biliary tract cancer, and osteoporosis in the disease MR analysis. *Solobacterium uSGB 2890* increased the risks of lung cancer and asthma (Supplementary Table 8). These findings suggest that the microbiota may influence longevity by acting on disease risks.

The reverse MR analysis (testing the causal effect of longevity on saliva microbiome) identified 122 suggestive links with *p* < 0.05 and 16 of them with *p* < 0.01 (Fig. 3b; Supplementary Table 7). For example, longevity was correlated with higher abundances of *Veillonellaceae F0422 uSGB 3578* (β = 0.024, *p* = 0.0095) whereas lower abundances of genus *Aggregatibacter* (β = − 0.023, *p* = 0.013) and a handful of its species such as *A. segnis and A. sp000466335,* family Porphyromonadaceae and its several species such as *P. sp000467855* and *P. endodontalis*. *Veillonellaceae* might be better adapted to an environment with lower saliva flow^10^, and could produce carbon dioxide from lactate, which might be an important source when elderly people do not eat as much^41^. Longevous individuals exhibited a lower abundance of phylum Fusobacteria (β =  − 0.023, *p* = 0.018) but higher Firmicutes (β = 0.019, *p* = 0.039), consistent with the above gut MR findings. Additionally, longevity was found to be negatively associated with the microbial function/module MF0025:tryptophan degradation for both saliva and gut, in agreement with a previous report that aging and the aging-associated decline in protein homeostasis could be delayed by inhibition of tryptophan degradation^42^.

### Causalities between the tongue microbiome with longevity

**Figure 4:**
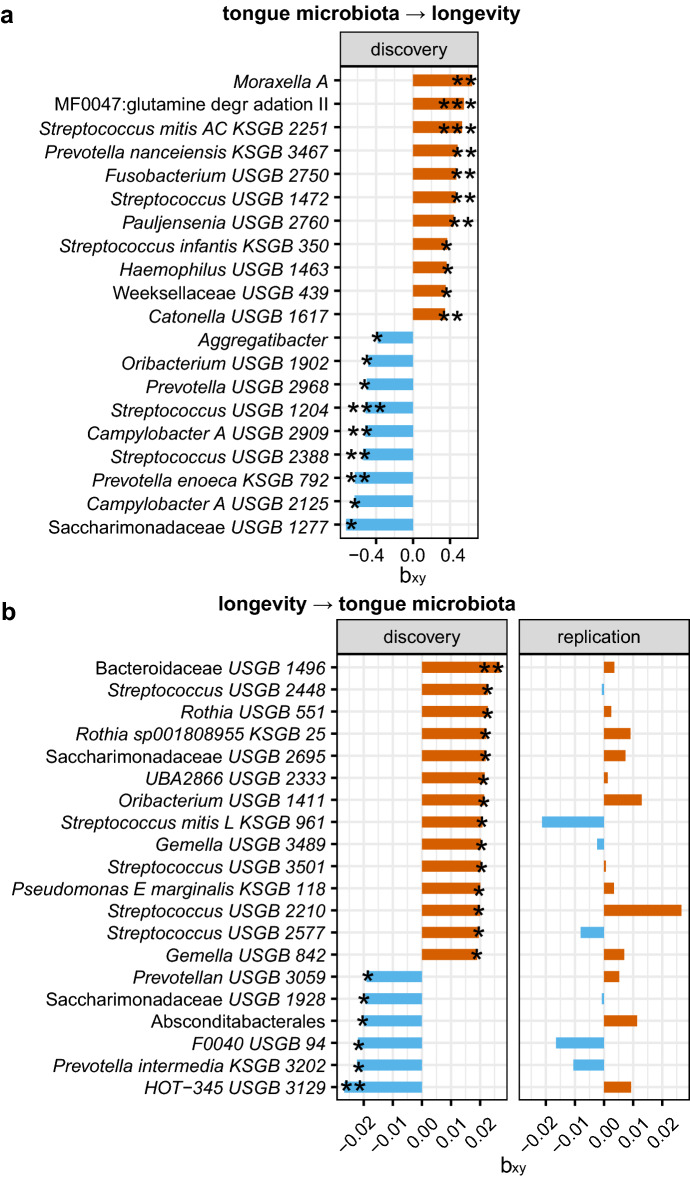
Bidirectional two-sample MR analyses identified causal relationships between the tongue dorsum microbiome and longevity. (**a**) The barplot represented the top 20 causal effects of specific tongue dorsum microbial features on longevity (*p* < 0.05), as estimated using the GCTA-GSMR approach in the forward MR analysis. The MR effect size estimate (bxy) was plotted. (**b**) The barplot represented the top 20 causal effects of longevity on tongue dorsum microbial features in the reverse MR analysis (*p* < 0.01). Discovery used the longevity phase I dataset (N = 4477) as exposure. Replication used the longevity phase II dataset (N = 6548) as exposure. Both used the gut microbiome dataset (N = 1539) as the outcome. *p < 0.05, **p < 0.01, and ***p < 0.001. Unclassified species-level cluster denotes uSGB, known species-level clusters denote kSGB and MF denotes microbial function/module.

We also examined the causal relationships between the tongue microbiome and longevity. 73 tongue microbiome features linked to longevity (Supplementary Table 9), among which 12 links with *p* < 0.01 were presented in Fig. 4a. For example, *Streptococcus mitis AC* (β = 0.531, *p* = 0.0005)*, Streptococcus uSGB 1472* (β = 0.464, *p* = 0.005), *Prevotella nanceiensis* (β = 0.481, *p* = 0.0048), and *Catonella uSGB 1617* (β = 0.346, *p* = 0.0064) were found to be positively correlated with longevity. However, *Streptococcus uSGB 1204* (β =  − 0.513, *p* = 0.00096), *Prevotella enoeca* (β =  − 0.629, *p* = 0.0019) and *Campylobacter A uSGB 2909* (β =  − 0.517, *p* = 0.006) were negatively correlated with longevity. Moreover, tongue dorsum microbial pathway MF0047: glutamine degradation II promoted longevity (β = 0.551, *p* = 0.00098), consistent with the above gut MR result that longevity was positively linked to fecal MF0047: glutamine degradation II. *Aggregatibacter* (β =  − 0.383, *p* = 0.017) and *A. segnis* showed negative links with longevity, in line with the longevity influences on *Aggregatibacter* spp. as identified in the saliva MR result*.*

Further, we also found 29 causal effects of longevity on the tongue dorsum microbiome (*p* < 0.05). Longevity was genetically linked to decreased abundances of *Prevotella intermedia* that was associated with increased risks of CRC^43^ and severe asthma^44^, *HOT-345 uSGB 3129*, *F0040 uSGB 94,* but increased abundances of *Oribacterium uSGB 1411, Saccharimonadaceae UBA2866 uSGB 2333, Rothia uSGB 551, Streptococcus uSGB 2448, Bacteroidaceae uSGB 1496* (Fig. 4b, Supplementary Table 9).

### Longevity associated with microbial α-diversity

**Figure 5:**
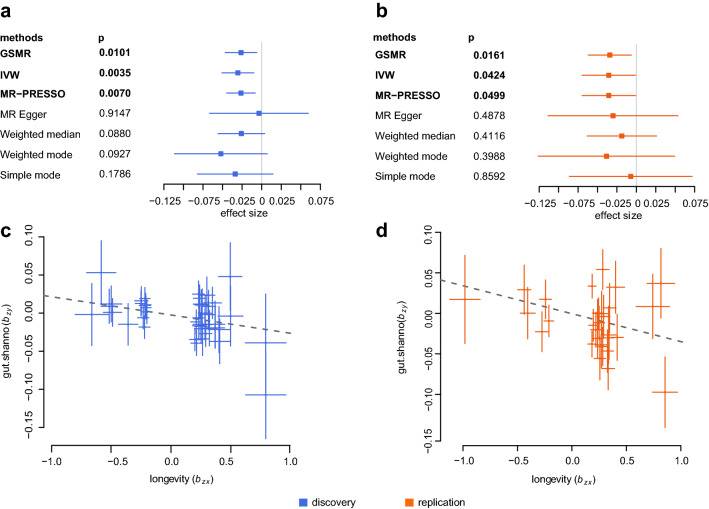
Bidirectional two-sample MR inference for the microbial α-diversity and longevity. (**a,b**). Forest plots represented the MR estimates and 95% CI values of the causal effects of longevity on microbial alpha diversity, as estimated in the discovery ((**a**), blue) and replication ((**b**), red) stages, respectively. (**c,d**) plotted the instruments of longevity phase I (discovery, (**c**)) and phase II (replication, (**d**)) datasets, respectively, showing the causal effect of longevity on lower gut microbial α-diversity. The x-axes in (**c,d**) show the SNP-exposure effect and the y-axes show the SNP-outcome effect. Error bars in (**c,d**) represent the standard errors.

We further investigate whether there is a potential correlation between microbial α-diversity and longevity. We identified 7, 21, and 11 independent host genetic variants associated with microbial α-diversity (Shannon diversity index) for the gut, saliva, and tongue dorsum at *p* < 10^–5^, respectively (Supplementary Table 10). Using these genetic variants as instrumental variables (IVs), we performed two-sample BMR analyses and observed no significant influences of microbial diversity across all three body niches on longevity (Supplementary Table 11). However, the reverse MR inference showed that genetically longevous individuals exhibited a lower Shannon diversity in the gut (Fig. 5). This causal relationship was robust when using two different longevity datasets as exposures (Fig. 5). Unexpectedly, this finding was in contrast to the previous papers^45^.

## Discussion

This is the first study using the MR approach to reveal causal relationships between the human microbiome (gut and oral) and longevity. We provided a good repository of resources containing a large number of potential correlations between the gut-, salivary-, tongue dorsum- microbiome, and longevity using bidirectional two-sample MR analyses. Those specific microbes that were identified significantly associated with longevity could be potential causal targets for study on healthy aging^8^.

Some of the identified longevity-associated microbes were found to correlate with diseases, indicating these microbes may affect longevity by mediating the risks of diseases. Coriobacteriaceae was significantly reduced in heart failure patients with reduced ejection fraction and cardiomyopathy^46^, suggesting this family may contribute to longevity by reducing the risk of cardiovascular diseases. Phylum Tenericutes was more abundant in the healthy group than in the MetS group in a Korean study^47^ and decreased the risk of lung cancer in our previous MR analysis^21^. *Oxalobacter* could potentially metabolize oxalate which hurts kidney functions^48,49^, was related to pro-longevity maybe also through reducing the risk of rheumatoid arthritis (RA) as reported in an MR study^22^. The probiotic *Lactobacillus amylovorus* altering body adiposity^28^ positively correlated with longevity, whereas the CRC pathogen *Fusobacterium nucleatum*^30^ negatively correlated with longevity. Consistently, our diseases’ two-sample MR analysis also supported a “microbiota—disease—longevity” axis (Supplementary Table 8). For example, the gut Tenericutes that decreased the risk of lung cancer in our previous MR analysis^21^ promoted longevity. The salivary *Oribacterium sinus and Pauljensenia uSGB 2326* that were negatively associated with congestive heart failure promoted longevity. Some other pro-longevity features such as *Streptococcus infantis I* and *Lancefieldella uSGB 1105* were also negatively associated with peripheral artery disease that caused a crude five-year death rate of 33.2%^50^. These findings further help us to understand the interactions among microbiota, disease, and longevity.

We not only excavated the specific microbiota that may affect longevity but also investigated which microbes the genetically predicted longevous individuals tended to colonize. Here, longevity was genetically related to a higher abundance of *Prevotella* but a lower abundance of *Bacteroides*. *Prevotella* was associated with plant-rich diets (high levels of complex carbohydrates and fruit and vegetable intake), whereas *Bacteroides* was dominated by the high-fat, high-sodium “westernized” diet^51,52^, indicating that long-lived individuals may genetically tend to prefer healthier diets with higher intakes of vegetables and lower intakes of chloride, sodium, and saturated fat food that caused higher *Prevotella* and lower *Bacteroides* abundances,respectively^53,54^.

There are two major limitations in this study. First, the identified causalities didn’t reach a multiple-testing corrected significance given that we performed MR tests on thousands of microbial features. Despite this, we were still able to replicate 17 of the 78 identified causalities from longevity to the gut microbiome by using an independent dataset as validation, supporting the robustness of these MR results. Our data-driven MR results also supported and confirmed some previously reported observational correlations or animal experimental results. For example, observational studies showed that longevity was associated with a lower abundance of *Bacteroides*^6,34^ but higher abundances of Firmicutes and Tenericutes^34^, *Corprococcus*^6,55^. The positive correlation between longevity and microbial pathway MF0047: glutamine degradation II in both tongue dorsum (β = 0.551, *p* = 0.00098) and gut (β = 0.021, p = 0.00022) agreed with previous findings that glutamine-supplemented mice also showed remarkably a full one-third extension of lifespan^56^. Longevity was positively correlated with the gut microbial pathway hydrogen metabolism, which extends the lifespan of *C. elegans* by reducing reactive oxygen species^57^. Second, the lack of individual-level data prevented us from directly comparing the contributions of the microbiome and other host factors (economic/behavioral/environmental) to longevity. Our previous study has reported that the most influential factors related to longevity included sex, education level, career status, smoking, drinking, and diseases such as hypertension, type 2 diabetes mellitus, cardiovascular disease, dyslipidemia, gastroenteric ulcer, arthritis or cholelithiasis^33^. These factors may have effects on longevity independent of microbiome profiles. We hope to collect more individual-level data including the microbiome, genome, socioeconomic, behavioral, and environmental factors in the longevity cohort to comprehensively explore the impact of these factors on longevity in the future.

There were two important points to notice in this and future related research. One is that causalities of the gut microbiome and longevity generally exhibited negative correlations among Chinese and European populations, which may be partly attributed to geography or ethnicity-specific variations in the microbiome compositions that could be affected by diets, lifestyles, and genetic backgrounds^58,59^. For example, Verrucomicrobia and its genus *Akkermansia* negatively correlated with longevity in the Netherlands but positively associated with longevity in the Chinese. *Akkermansia muciniphila* is an emerging probiotic that has been reported to promote longevity and slow down the aging process^60,61^. *Lactobacillus* and *Streptococcus* negatively contributed to longevity in the Chinese but showed no associations in the Europeans. The genetically predicted longevous individuals tend to exhibit higher abundances of *Prevotella* and *Paraprevotella* in the Chinese but not in the Europeans which was consistent with the observational study^25^, suggesting the higher prevalence of *Prevotella* in Asian countries might be determined by ancestral genomes in addition to the diet. Future studies in cohorts of different ethnicities with larger sample sizes were called for to further substantiate these findings.

Another is to distinguish the associations of microbiota across different body sites with longevity. Longevous individuals causally exhibited a lower abundance of phylum Fusobacteria and its species (*F. nucleatum*^30^ and *F. varium*^21^) in both gut and saliva. However, some bacteria that decreased longevity in the gut appeared to increase longevity in the mouth, where they normally belong. For example, *Streptococcus* was negatively associated with longevity in the gut, whereas, *Streptococcus mitis*, *Streptococcus uSGB 1603*, *uSGB 1349*, *uSGB 2667*, and *Streptococcus infantis G* were positively associated with longevity in the saliva. These findings also implied the similarities and differences in the gut and oral microbial compositions and diversities^10,26,62^. The transmission and colonization of commensal microbes among different body sites would need to be monitored for a long and healthy life.

In summary, our MR analysis illustrates the value of human genetic information to help prioritize human microbiome features for aging-related mechanistic studies and clinical trials.

## Methods

### Microbiome cohort

The microbiome data in this study were originally from the 4D-SZ cohort^21,25,26,32,63^, which is a multi-omics cohort, and all individuals in which were enlisted for the whole genome sequencing (WGS) and whole metagenomic sequencing (WMS).

We obtained GWAS summary statistics of 500 gut microbial features (401 taxa and 99 pathways) in 1539 individuals as previously detailly described^21^. Briefly, the gut microbial taxonomy profiles were generated by aligning high-quality sequencing reads to the integrated gene catalog (IGC)^64^ of gut using SOAP2 with the criterion of identity ≥ 95% and were next determined based on the aligned gene abundances. Gut metabolic modules or pathways profiles were built through an extensive review of the literature and metabolic databases, inclusive of MetaCyc^65^ and KEGG^66^, followed by expert curation and delineation of modules and alternative pathways.

For oral microbiome, we obtained 1665 tongue dorsum microbial features (1583 taxa and 82 pathways) in 2017 individuals and 1770 salivary microbial features (1685 taxa and 85 pathways) in 1915 individuals with available GWAS summary statistics as detailly described here^26^. Briefly, the oral microbial taxonomy profiles were constructed by aligning reads to a previously well-constructed high-quality oral genome catalog^63^ and were next determined based on the aligned contig abundances. Oral metabolic modules or pathways profiles were built through an extensive review of the literature and metabolic databases, inclusive of MetaCyc^65^ and KEGG^66^, followed by expert curation and delineation of modules and alternative pathways. All those GWAS summary statistics included ~ 10 M common variants (MAF > 0.005) in this 4D-SZ cohort.

### Longevity cohort

The longevity GWAS data in this study comes from the Chinese Longitudinal Healthy Longevity Surveys (CLHLS), which were conducted in 1998, 2000, 2002, 2005, 2008, 2011, and 2014 in a randomly selected half of the counties and cities in 22 out of 31 provinces in China^27,33,67,68^. Two longevity datasets were included in the MR analysis. The longevity phase I dataset^27^ was the main longevity dataset that included 4477 samples (2178 centenarians and 2299 middle-aged controls) with 5.6 M imputed genotypes. The longevity phase II dataset^33^ contained 287 K longevity candidate variants in 6548 individuals that were independent and had no overlapping samples with the phase I dataset. Due to the longevity phase II dataset only containing longevity candidate variants, we took it as the replication exposure dataset to increase power when calculating the MR from longevity to microbiome direction.

### Two-sample MR design

We applied a bidirectional two-sample MR strategy: (1) In the forward direction, the GWAS summary statistics of gut and oral microbiota obtained from 4D-SZ cohort (500 gut microbial features for 1539 individuals, 1665 tongue dorsum microbial features for 2017 individuals and 1770 salivary microbial features for 1915 individuals; all individuals with high-depth whole genome and metagenome sequencing) were used as sample exposure, and the GWAS summary statistics from longevity phase I dataset (N = 4477 for 5.6 M genome-wide imputed variants) were used as sample outcome, to identify the causal effects of the microbiota on longevity; (2) In the reverse direction, the GWAS summary statistics from longevity phase I project were used as discovery sample exposure, and significant candidate longevity genes from longevity phase II dataset (N = 6548 independent and no overlapping samples with phase I project for 287 K candidate variants) were used as replication sample exposure, and the gut and oral microbiota obtained from 4D-SZ cohort were used as sample outcome, to identify the causal effects of longevity on the microbiota.

### Instrumental variable selection

For each genome-wide association result of microbial features and host longevity phenotype, we selected genetic variants that showed association at *p* < 1 × 10^−5^ and then performed the linkage disequilibrium (LD) estimation with a threshold of LD r^2^ < 0.1 for clumping analysis to get independent genetic variants as IVs. As previously described^21^, the *p*-value threshold of 1 × 10^−5^ was used for the selection of genetic IVs associated with microbial features by maximizing the strength of genetic instruments and the amount of the average genetic variance explained by the genetic predictors in an independent sample. Therefore, we used a more liberal threshold of *p* < 1 × 10^–5^ to select the instruments for microbial features, and the instrumental mean F statistics reached 51.4 for the gut microbiome, 22 for the tongue dorsum microbiome, and 18 for the salivary microbiome (all greater than 10) that indicates strong instruments, consistent with our previous study. We assessed the IV assumptions^69^ by directly testing the association of the selected IVs with a range of potential confounders. In this study, we selected potential confounders comprised of 220 phenotypes in BBJ^70^, including diseases such as type 2 diabetes, and colorectal cancer and metabolic traits such as body mass index (BMI), high-density lipoprotein (HDL), low-density lipoprotein (LDL), and triglycerides that were commonly reported linked to the microbiome. As shown in Supplementary Table 12, we identified an average of 45 IVs for the 500 gut microbial features, 63.6% (= 318/500) microbial features exhibited no IVs associated with any of the 220 potential confounders (p > 10^−5^) and 28.6% microbial features exhibited only one genetic variant that was associated with one of the 220 potential confounders (p < 10^−5^ according to the *p*-value cut-off definition of significant IVs). Altogether, 92.2% microbial features had no more than 1 potential confounder-associated IV given a mean of 45 IVs for each microbial feature. Similarly, for the 1770 salivary microbial features (an average of 39 IVs for each feature), 69.72% (= 1234/1770) microbial features exhibited no IVs associated with any of the 220 potential confounders and 94.29% microbial features had no more than 1 potential confounder-associated IV. These results indicated the MR analyses in this study basically met the instrumental variable assumptions.

For consistency, we used the same threshold and procedure for selecting genetic IVs of longevity traits in both the longevity discovery and the replication cohort. We retained a total of 45 IVs (F statistics = 59.2) from the longevity exposure cohort and 30 IVs (F statistics = 46.7) from the longevity replication cohort for the MR analysis. A list of genetic instruments could be checked in Supplementary Fig. 2.

### Two-sample MR analysis

The bidirectional two-sample MR analysis was performed by applying the GCTA-GSMR (Generalised Summary-data-based Mendelian Randomization) method, which excludes SNPs that show evidence of pleiotropic effects by the heterogeneity in dependent instruments outlier analysis (HEIDI-outlier test, 0.01). We therefore estimated a causal association between the exposure and the outcomes using the GSMR method^71^ as our principal MR analytical approach.

We subsequently performed a sensitivity and validation analysis for each MR using five other methods (inverse-variance weighting (IVW)^72,73^, MR-Egger regression^74^, weighted median^75^, mode-based estimate (MBE)^76^ including Simple mode and Weighted mode) implemented in the “TwoSampleMR” R package. A consistent effect across the four methods is less likely to be a false positive. We used the MR–Egger intercept and MR-PRESSO global test to test for the presence of directional pleiotropy. MR tests were considered statistically significant when *p* < 0.05.

### Two-sample MR analysis for European populations

For comparison, we also performed a two-sample MR analysis to investigate causal effects between microbial features and longevity in European populations. GWAS summary statistics of the gut microbiome were obtained from three published studies involving MiBioGen (mainly European populations)^22^, Dutch^35^, and Finnish^36^ cohorts. The MiBioGen performed a meta-analysis of the gut microbiota GWAS in 18,340 participants with 13,266 European ancestry^22^. The Dutch microbial GWAS used metagenomic sequencing data on 7738 individuals from the northern Netherlands^35^. The Finnish microbial GWAS was performed on 5959 genotyped individuals with matched gut microbial shotgun metagenomes in the Finnish cohort^36^. GWAS summary statistics of longevity was from the largest meta-analysis of human longevity GWAS of European ancestry^37^. We used the 99th percentile longevity data whose individuals lived beyond the 99th (N = 3484) percentile, which was comparable to the Chinese longevity study with centenarians (N = 2187) as cases. For consistency, genetic variants with *p* < 1 × 10^−5^ and LD r^2^ < 0.1 were selected as instrumental variables and the GCTA-GSMR approach was used for MR analysis. The 1kgenome Phase 3 dataset with only European population ancestry is used as the reference panel for LD clumping.

### Two-sample MR analysis for diseases in Japan Biobank

To investigate the causal effect of microbial features on diseases, we performed a bidirectional two-sample MR analysis using summary statistics of oral microbiota from our cohort and disease information from Biobank Japan. We downloaded both summary statistics data for 42 diseases from Biobank Japan^77^ (http://jenger.riken.jp/en/result). The bidirectional two-sample MR analysis was performed by applying the GCTA-GSMR^71^ using the HEIDI-outlier analysis to remove horizontal pleiotropic SNPs. For consistency, genetic variants with *p* < 1 × 10^−5^ and LD r^2^ < 0.1 were selected as instrumental variables for oral microbiota in our cohort. For disease exposures, SNP instruments were selected at a genome-wide significant threshold (*p* < 5 × 10^–8^) in the Japan Biobank study.

## Supplementary Information

Supplementary Figures.

Supplementary Table 1.

Supplementary Table 2.

Supplementary Table 3.

Supplementary Table 4.

Supplementary Table 5.

Supplementary Table 6.

Supplementary Table 7.

Supplementary Table 8.

Supplementary Table 9.

Supplementary Table 10.

Supplementary Table 11.

Supplementary Table 12.

## Data Availability

All statistical results were available in supplementary tables. The summary statistics including the associations between host genetics and the gut microbiomes, host genetics and metabolites are publicly available from https://ftp.cngb.org/pub/CNSA/data2/CNP0000794/. The summary statistics including the associations between host genetics and tongue dorsum microbiome, host genetics and saliva microbiome are publicly available from https://db.cngb.org/search/project/CNP0001664. The release of these summary statistics data was approved by the Ministry of Science and Technology of China (Project ID: 2020BAT1137; 2021BAT1539). The summary statistics of microbial features from the MiBioGen cohort are available at www.mibiogen.org. The summary statistics of microbial features from the Finnish cohort are available via the NHGRI-EBI GWAS catalog https://www.ebi.ac.uk/gwas/studies/GCST90032172. The summary statistics of microbial features from the Dutch cohort are available at https://molgenis26.gcc.rug.nl/downloads/dutchmicrobiomeproject/summarystats/taxa. The full meta-analyses summary statistics of longevity are available for download at www.longevitygenomics.org/downloads.
