# Supplementary material for: Mendelian randomization analyses reveal causal relationships between the human microbiome and longevity
Source: Sci Rep. 2023 Mar 29;13:5127. doi: 10.1038/s41598-023-31115-8 (PMC10052271; doi:10.1038/s41598-023-31115-8)
Supplement: Supplementary file 1 — Supplementary Figures. [file 41598_2023_31115_MOESM1_ESM.docx]

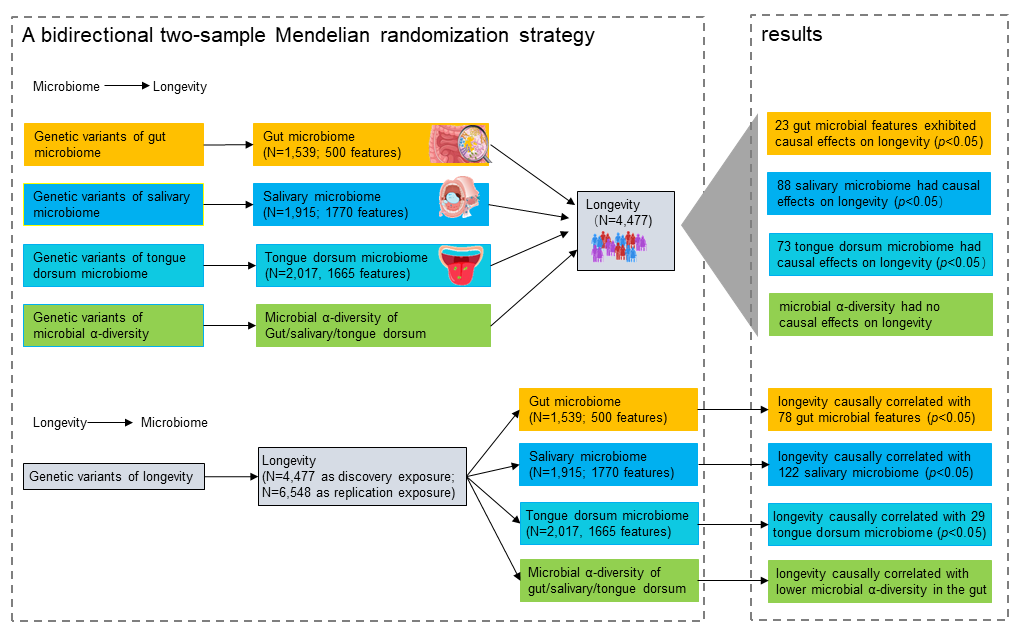


**Supplementary Fig. 1. An overview of the study design and results.** The left box of the figure showed the workflow of the bidirectional two-sample Mendelian randomization (MR) analysis. The forward MR was performed using GWAS summary statistics of the gut-, salivary-, tongue dorsum- microbiome, and microbial α-diversity from the 4D-SZ cohort (a total of N=2,984 for high-depth whole genome and metagenome sequencing) as exposures data and GWAS of the longevity from the CLHLS phase I (N=4,477) as the outcome data, to identify the causal effects of microbiota on longevity. The reverse MR was performed using longevity GWAS of the CLHLS phase I dataset (N=4,477) as discovery sample exposure and longevity GWAS of the CLHLS phase II dataset (N=6,548 for 278K candidate variants, independent and no overlapping samples with the phase I dataset) as replication sample exposure, together with gut-, salivary-, tongue dorsum- microbiome, and microbial α-diversity as outcomes, to identify the causal effects of longevity on the microbiota. The right box presented the corresponding results based on the MR analyses. Please see the Main texts for details.


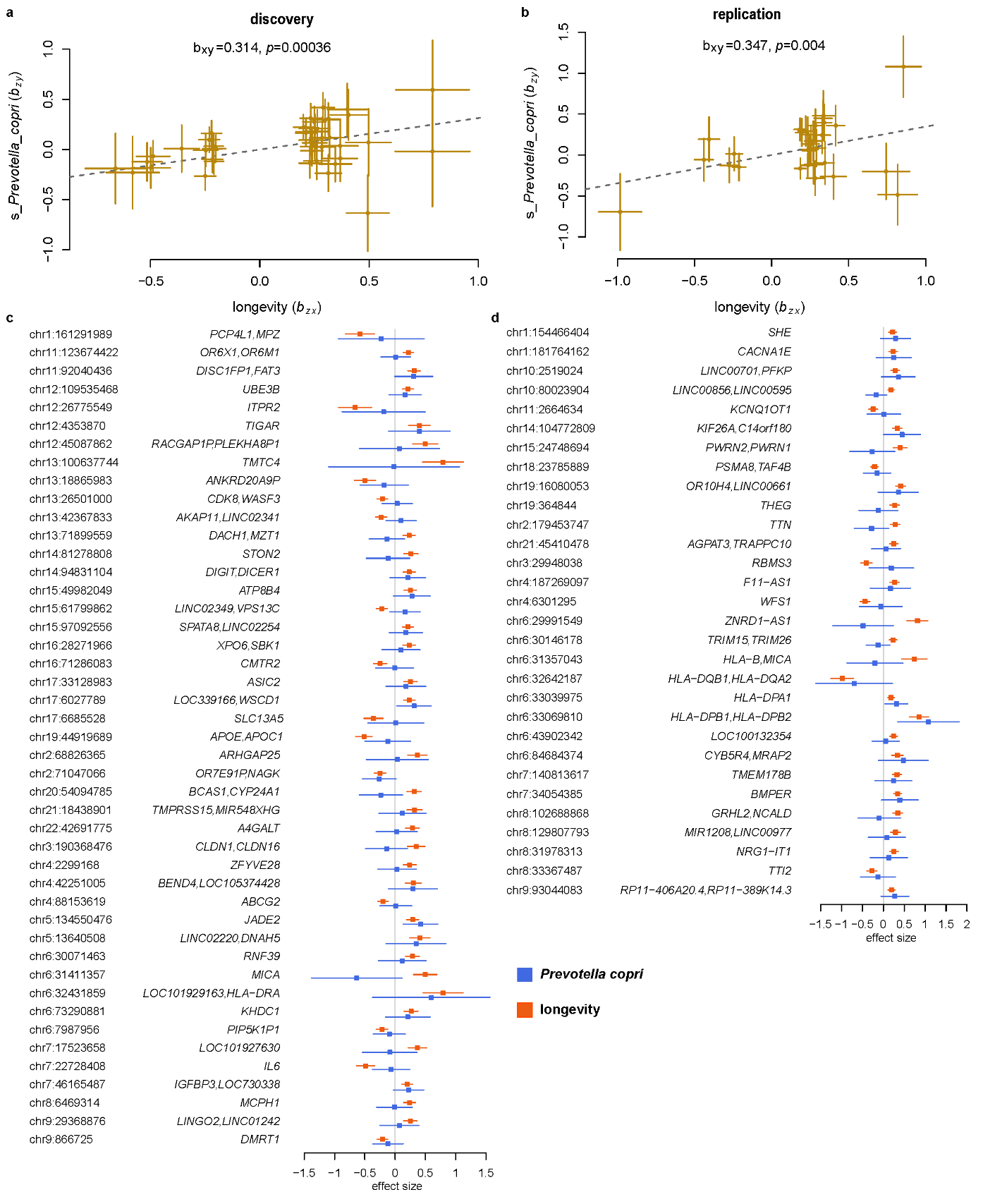


**Supplementary Fig. 2. Longevity is causally associated with a higher abundance of *Prevotella* species such as *P. copri.*** The causal effect of longevity on *P. copri* was identified when using the longevity phase I dataset as discovery exposure (**a**) and also well replicated when using the phase II dataset as replication exposure (**b**), using the GSMR method. **c** showed the instrumental variables (IVs) and their associated *p* with longevity and *P. copri* when using the longevity phase I dataset as discovery exposure for analysis. **d** showed the instrumental variables (IVs) and their associated *p* with longevity and *P. copri* when using the longevity phase II dataset as replication exposure for analysis***.***

***
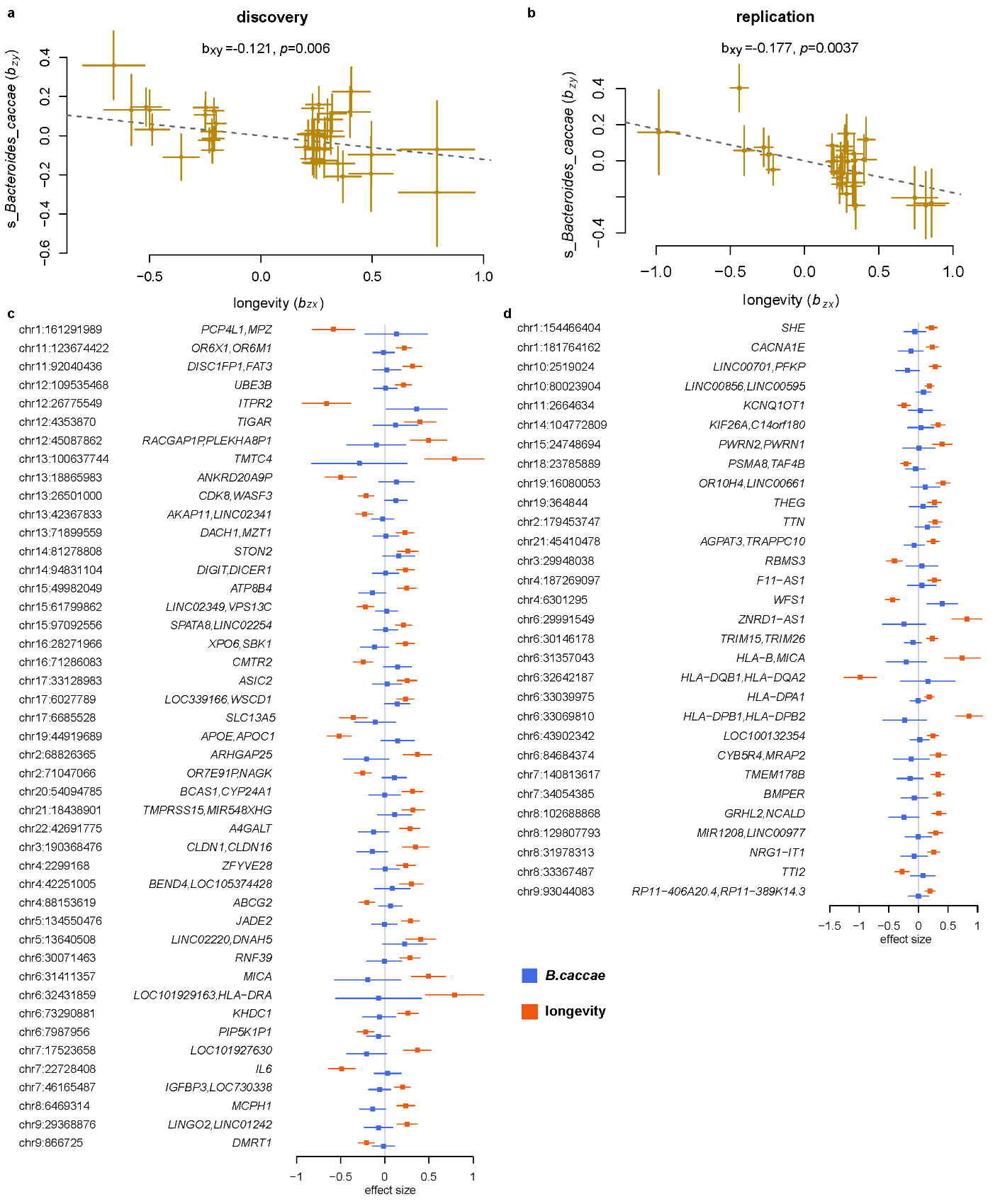
***

**Supplementary Fig. 3. Longevity is causally associated with a lower abundance of *Bacteroides* species such as *B. caccae.*** The causal effect of longevity on *B. caccae* was identified when using the longevity phase I dataset as discovery exposure (**a**) and also well replicated when using the phase II dataset as replication exposure (**b**), using the GSMR method. **c** showed the instrumental variables (IVs) and their associated *p* with longevity and *B. caccae* when using the longevity phase I dataset as discovery exposure for analysis. **d** showed the instrumental variables (IVs) and their associated *p* with longevity and *B. caccae* when using the longevity phase II dataset as replication exposure for analysis***.***


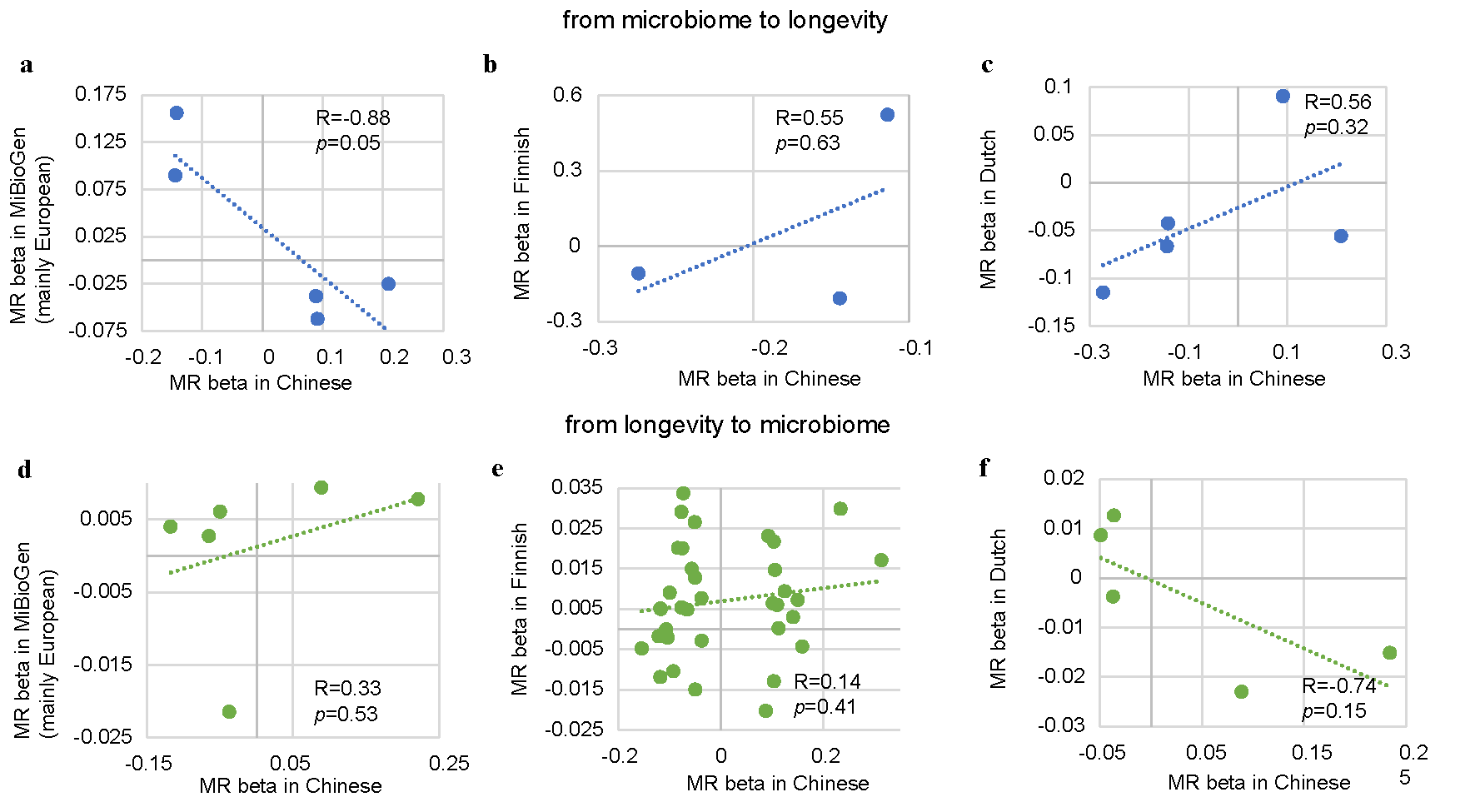


**Supplementary Fig. 4. Comparisons of Chinese-significant causalities with that of European populations from MiBioGen (mainly European), Finnish and Dutch cohorts. a-c** represented the causalities from gut microbiome to longevity (blue dots) by comparing the effect sizes of Chinese-significant causalities (*p*<0.05) with that of MiBioGen (mainly European, **a**), Finnish(**b**), and Dutch(**c**), respectively. **d-f** represented the causalities from longevity to gut microbiome (green dots) by comparing the effect sizes of Chinese-significant causalities (*p*<0.05) with that of MiBioGen (mainly European, **d**), Finnish(**e**), and Dutch(**f**), respectively. Spearman test was used with correlation coefficients and *p*-value marked.


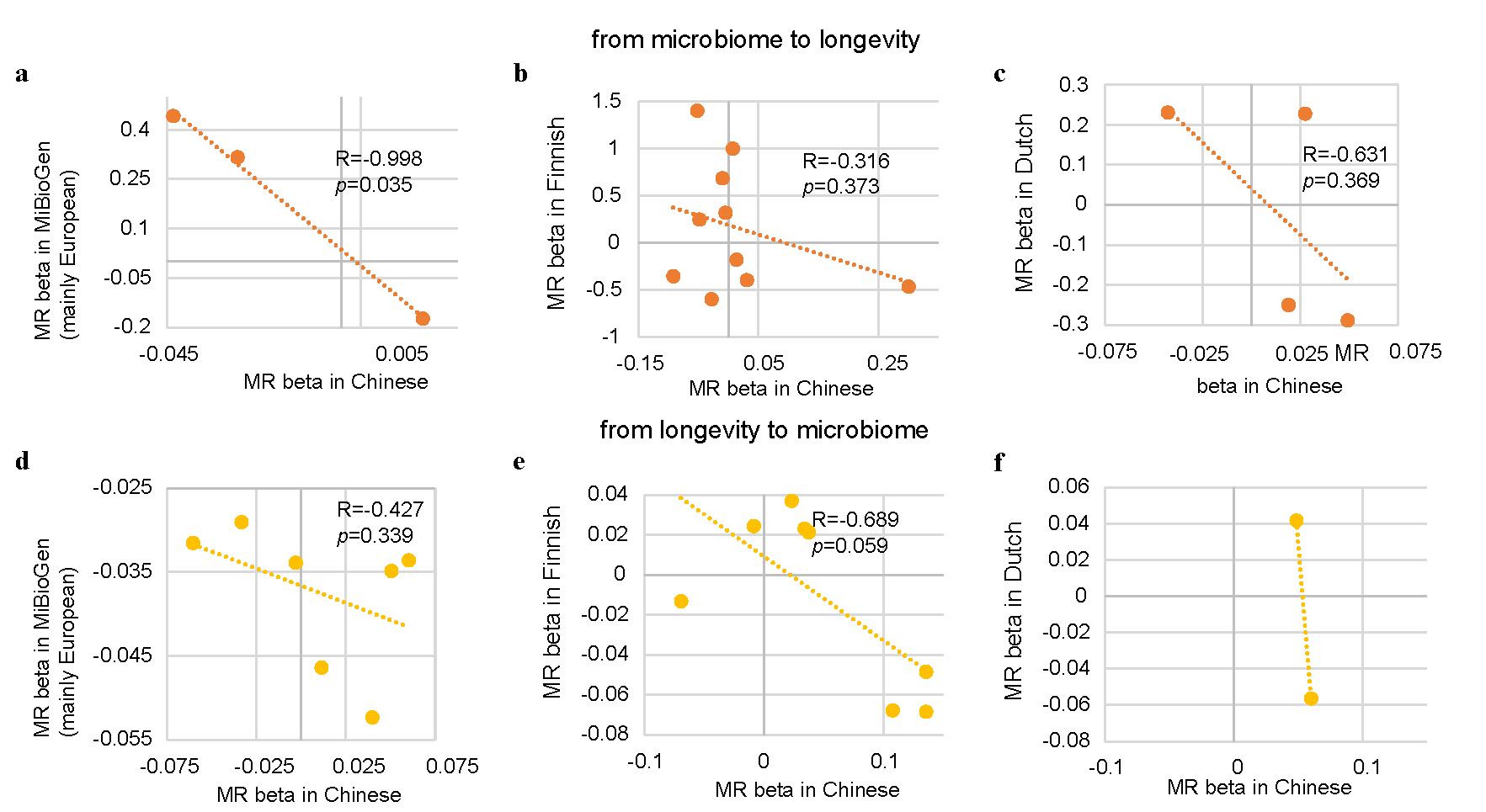


**Supplementary Fig. 5. Comparisons of European-significant causalities with that of Chinese.** These causalities between gut microbiome and longevity were significant in MiBioGen (mainly European), Finnish, and Dutch cohorts, respectively (*p*<0.05). We compared the beta effect sizes of these causalities among the corresponding European cohort and Chinese, using the Spearman test with correlation coefficients and *p*-value marked. **a-c** represented the causalities from gut microbiome to longevity (red dots) and **d-f** represented the causalities from longevity to gut microbiome (yellow dots).
